# Gut Microbiota Alterations in Alzheimer’s Disease: Relation with Cognitive Impairment and Mediterranean Lifestyle

**DOI:** 10.3390/microorganisms12102046

**Published:** 2024-10-10

**Authors:** David Mateo, Nerea Carrión, Cristian Cabrera, Luis Heredia, Montse Marquès, Eva Forcadell-Ferreres, Maria Pino, Josep Zaragoza, Alfons Moral, Lluís Cavallé, José M. González-de-Echávarri, Paloma Vicens, José L. Domingo, Margarita Torrente

**Affiliations:** 1 Laboratory of Toxicology and Environmental Health (LSTM), Centre for Environmental, Food and Toxicological Technology (TECNATOX), Universitat Rovira i Virgili, 43201 Reus, Spain; david.mateo@urv.cat (D.M.); nerea.carrion@urv.cat (N.C.); cristian.cabrera@alumni.urv.cat (C.C.); luis.heredia@urv.cat (L.H.); mmbehq@cid.csic.es (M.M.); paloma.vicens@gmail.com (P.V.); joseluis.domingo@urv.cat (J.L.D.); 2 Institut d’Investigació Sanitària Pere Virgili (IISPV), 43204 Reus, Spain; 3 Department of Psychology, Faculty of Education Sciences and Psychology, Universitat Rovira i Virgili, 43007 Tarragona, Spain; 4 Institute Lerin Neurocognitive, Alzheimer and Other Neurocognitive Disorders Association, 43205 Reus, Spain; 5 Research Center for Behaviour Assessment (CRAMC), Faculty of Education Sciences and Psychology, Universitat Rovira i Virgili, 43007 Tarragona, Spain; 6 Department of Research Methods and Diagnosis in Education, Universidad Internacional de la Rioja, 26006 Logroño, Spain; 7 Neurology, Hospital Verge de la Cinta de Tortosa, 43500 Tortosa, Spain; eforcadellf.ebre.ics@gencat.cat (E.F.-F.); jzaragoza.ebre.ics@gencat.cat (J.Z.); 8 Cognitive Impairment Unit, University Hospital Sant Joan de Reus, 43204 Reus, Spain; maria.pino@salutsantjoan.cat; 9 Neurology, Xarxa Santa Tecla, 43003 Tarragona, Spain; amoral@xarxatecla.cat; 10 Neurology, University Hospital Joan XXIII, 43005 Tarragona, Spain; llmoreno.gipss@gencat.cat (L.C.); jmgonzalez.hj23.ics@gencat.cat (J.M.G.-d.-E.)

**Keywords:** gut microbiota, Alzheimer’s disease, dementia, cognition, microbial diversity

## Abstract

Recently, an association between dysbiosis of the gut microbiota (GMB) and the development of several diseases, such as Alzheimer’s disease (AD), has been proposed. Dysbiosis involves changes in microbial diversity influenced by environmental factors, like diet or lifestyle. In this study, we investigated the role of GMB parameters in Spanish AD patients, assessing the impact of adherence to the Mediterranean lifestyle (ML), as well as to characterize these parameters in relation to neuropsychological, neuropsychiatric, emotional, and functionality parameters. A case–control study was conducted to investigate the association between the composition of the GMB and cognitive, emotional, neuropsychiatric, and functionality status in Spanish AD patients, along with a shotgun metagenomics approach. Richness and alpha-diversity were significantly lower in the AD group compared to the controls. PERMANOVA and ANOSIM tests of Bray–Curtis dissimilarity, Aitchison distance, and Jaccard similarity did not showed significant differences in beta-diversity between the two groups. Moreover, associations between various phyla of the AD group and orientation performance, food consumption, and activities of daily living were identified. Dysbiosis observed in Spanish AD patients is characterized by reductions in richness and alpha-diversity, alongside alterations in GMB composition, which may be linked to adherence to the ML and cognitive and functionality symptoms.

## 1. Introduction

The World Health Organization (WHO) defines Alzheimer’s disease (AD) as a neurodegenerative disorder characterized by a progressive impairment of cognitive function [1]. Currently, the etiological mechanism of AD has not yet been fully elucidated. However, studies report evidence of a specific neuropathological profile [2] formed by the deposition in extracellular plaques of amyloid-β (Aβ) protein and intraneuronal neurofibrillary tangles of tau protein (NFTs) [3]. AD accounts for around 50% to 75% of the total number of cases of dementia [4]. Increasing life expectancy is leading to an aging population, increasing the prevalence of very serious age-related pathologies such as AD among the elderly. In Europe, the prevalence of AD is 0.97% among people aged 65–74 years, 7.66% among people aged 75–84 years, and 22.53% among people over 85 years old [5].

Recent studies have elucidated the crucial role of gut microbiota (GMB) in the pathogenesis of various diseases [6]. GMB are the microorganisms (bacteria, archaea, fungi, and viruses) inhabiting the digestive tract, being the major location of microbes in the human body (10 to 100 trillion) [7]. Emerging evidence suggests that GMB may significantly contribute to dementia pathogenesis [7,8,9] by modulating host-brain function via a recently discovered microbiota–gut–brain axis [10]. Regarding specifically the pathogenesis of AD, intestinal dysbiosis (an alteration in the normal commensal intestinal microbiome with an increase in pathogenic microbes) may lead to an increase in the permeability of the gut and blood–brain barriers, triggering the activation of immune responses and leading to an increased level of oxidative stress. This process facilitates the development of characteristic features of AD, including Aβ aggregation, oxidative stress, neuroinflammation, and insulin resistance [11]. A recent systematic review and meta-analysis showed a significant reduction in species diversity richness within the human AD gut microbiome [12]. This study revealed region-specific variations in the abundance of Bacteroides, which is among the most abundant groups of bacteria found in the human intestine, with higher levels observed in US cohorts compared to Chinese cohorts [12]. These inter-study disparities underscore the impact of diverse environmental factors on GMB composition, including aspects like ethnicity, demographics, lifestyle, and diet [13]. Notwithstanding, the existence of a distinctive GMB composition characteristic of AD remains to be determined, as well as whether the observed GMB alterations act as precursors, contributing to the initiation or progression of the disease, or if they are the result of its pathological processes.

Most risk factors associated with a higher susceptibility to AD, including age, gender, genetics, and family history, cannot be changed or eliminated. However, epidemiologic research has revealed the existence of potential modifiable risk—and protective factors—for AD and related dementias. Recently, The Lancet Commission on Dementia Prevention, Intervention and Care suggested that tackling modifiable risk factors such as low educational attainment in early life, mid-life hypertension, mid-life obesity, hearing loss, traumatic brain injury, excessive alcohol consumption, smoking, depression, physical inactivity, social isolation, diabetes mellitus, and air pollution could potentially prevent or delay up to 40% of dementia cases [14]. Most of these factors have been linked to GMB alterations [15], and several GMB-modulation strategies, such as diet and lifestyle choices, could currently serve as a crucial foundation for developing strategies to prevent or treat AD [12]. In fact, both are strongly related to GMB, being potentially modifiable [16]. One of the main factors influencing GMB throughout life is the diet [17]. In relation to this, the Mediterranean diet (MD) is a prime example of how healthy dietary patterns can be beneficial for gut health [18]. The MD, which is characterized by its richness in plant-based foods, healthy fats, ingestion of minimally processed foods, important consumption of fish, moderate intake of dairy products, and moderate consumption of wine, has been associated with reduced AD hallmarks (Aβ and NFTs) [19] and AD risk [20]. Indeed, the significance of the MD might transcend into a broader Mediterranean lifestyle (ML), which encompasses additional healthy lifestyle behaviors such as sociability, sleep, rest, and conviviality [21], all of which have also been related to the maintenance of a healthy aging brain [22]. These factors have also demonstrated the capacity to influence the composition and functionality of the GMB [23].

To the best of our knowledge, the GMB composition related to AD has not been previously investigated considering the particularities of the ML, especially in relation to the MD. In the present study, we hypothesized that AD patients with adherence to the ML will present differences in the studied microbiological parameters (richness, alpha-diversity, and beta-diversity) in comparison from those described in other countries with different diets and lifestyles. A case–control study was hereby performed to investigate the association between the composition of the GMB of the selected population, employing a shotgun metagenomics approach with neuropsychological, neuropsychiatric, and functionality tests.

## 2. Materials and Methods

### 2.1. Study Design

The current investigation has complied with the Declaration of Helsinki, and it was approved by the Ethics Research Committee (CEIm) of the Pere Virgili Institute for Health Research (IISPV, Ref. CEIM: 183/2020). This study is also included on the ClinicalTrials.gov website (ID: NCT05943925). Informed consent was obtained from all patients and their families before participating in the study.

### 2.2. Participants

Twenty-five AD patients and twenty-five age- and sex-matched healthy controls (HCs) were enrolled by recruiting centers from Tarragona County (Catalonia, Spain) from May 2021 to December 2022. This sample size was expected to be enough to detect differences in gut microbiota with sequencing techniques [9]. The participant centers included Verge de la Cinta Hospital, Xarxa Santa Tecla Hospital, Joan XXIII Hospital, and Lerín Neurocognitive Institute. Inclusion criteria for the AD group were as follows (CCs): (1) AD diagnosed by neurology service following NIA-AA 2011 criteria, (2) aged between 60 and 85 years old, and (3) Global Deterioration Scale Fast (GDS-FAST) of 4 or 5 [24]. Inclusion criteria for the control group were the following: (1) healthy individuals, and (2) between 60 and 85 years old. Exclusion criteria for both groups were the following: (1) diagnosis of or comorbidity with other neurological diseases, (2) use of antibiotics or corticosteroids in the previous 6 months before enrolling in the study, (3) immunosuppressor or immunostimulant treatment in the previous 6 months before providing the stool sample, (4) illnesses of the GI tract, (5) consuming large doses of commercial probiotics (greater than or equal to 108 cfu per organisms per day), and (6) illiteracy.

The recruitment centers selected potential candidates among their regular patients. Those candidates and their families who agreed to participate in the study signed their informed consent, being provided with the stool collection kit, and were scheduled for assessments within a maximum period of one week (see below).

### 2.3. Fecal Sample Collection and DNA Isolation

Fecal samples were collected at home with the Stool Nucleic Acid Collection and Preservation System kit (Norgen Biotek Corporation, Thorold, ON, Canada) three days before the interview. Participants stored the collection kits at room temperature—or in the refrigerator—and brought them on the day of the interview. Aliquots (~500 mg) were placed into Eppendorf tubes, frozen, and stored at −80 °C. One aliquot per participant was analyzed by the Centre for Omic Sciences (COS, Reus, Spain). DNA was extracted using the Fast Stool DNA Mini Kit (Qiagen, Germany) with a previous lysis of the sample (200 ± 30 mg) in 100–200 μL nuclease-free water at 95 °C, according to the kit instructions. The final DNA concentration and purification were determined by a Qubit 4.0 fluorometer and Qubit dsDNA HS Assay Kit (Thermo Fisher Scientific, Waltham, MA, USA).

### 2.4. Shotgun Metagenomics and Quality Control

Gut microbiome was analyzed by COS using a shotgun metagenomic approach. DNA was extracted from samples using the DNA Prep with Tagmentation kit according to the manufacturer’s protocol (Illumina, San Diego, CA, USA, catalog no. 20018705). Sequencing library concentration was determined by Qubit 4.0 fluorometer and Qubit dsDNA HS Assay Kit (Thermo Fisher Scientific, Waltham, MA, USA). In addition, sequencing library length was checked by Agilent TapeStation and Agilent High Sensitivity DNA kit (Agilent Technologies, Santa Clara, CA, USA). Sequencing libraries with concentration below 750 pM and any length out of 400–600 bp range were discarded. Final sequencing libraries were mixed at 750 bp and sequenced using the NextSeq 2000 sequencing system (Illumina, San Diego, CA, USA) as 2 × 150 bp paired-end reads. Samples below 7.5 million reads were discarded. Shotgun metagenomic reads were profiled for microbial species relative abundances by mapping them to several databases with Kraken2 [24]. Unidentified reads were discarded.

### 2.5. Exploratory Assessment

All participants were interviewed for approximately 1.5 h at the recruiting centers. During the interview, the following parameters were recorded: (1) demographic characteristics, such as age, laterality, sex, nationality, profession, years of education, civil status, and cohabitants at home; (2) the presence of risk factors, such as family history of AD, deafness, hypertension, dyslipidemia, diabetes mellitus, obesity, stroke, toxics exposure, smoking habits, and alcohol consumption; and (3) use of medication and/or supplements. Additionally, patients and their families also completed the Mediterranean Style Index test (MEDLIFE), a 28-item self-report diet questionnaire about how the patient follows the MD and Mediterranean habits [21].

### 2.6. Cognitive Functions and Emotional, Neuropsychiatric, and Functionality Assessment

Cognitive functions were evaluated by trained researchers. They included the following tests in order of administration: (1) cognitive area, which includes Temporary, Spatial and Personal Orientation from the Barcelona Test II (TO-BTII) [25], a comprehensive neuropsychological battery used to evaluate cognitive functions, which is widely used in Spanish-speaking populations, Mini Mental State Examination (MMSE) [26], Memory Impairment Screen (MIS) [27], Digit Span from the Barcelona Test II (DS) [25], Free and Cued Selective Reminding Test (FCSRT) [28], Trail Making Test A (TMT A) and B (TMT B) [29], Clock Drawing Test with Cacho et al. [30] correction (CDT) [31], the Copy of Simple and Semi-complex Construction Praxis subtest of the Barcelona Test II (CCPS-BTII) [25], Frontal Assessment Battery (FAB) [32], Cognitive Reserve Scale (CRS) [33], the C-form shortened version of the Boston Naming Test [34,35], and Categorial Evocation Fluency from the Barcelona Test II (CEF) [25]; (2) emotional area, which includes the Goldberg Anxiety and Depression Scale (GOLDBERG) [36] and the Life Events Questionnaire, adapted from the PREDIMED-PLUS study (LEQ) [37]; (3) neuropsychiatric area, which includes the neuropsychiatric symptomatology test from the BTII (NPBTII2 and NPEBTII) [25]; and (4) functionality, which includes The Daily Life Activities test from the BTII (ADL) [25]. A detailed description of the tests can be found in Supplementary Table S1.

### 2.7. Statistical Analysis

The statistical analysis was performed using the RStudio program. Continuous and categorical variables are expressed as mean and standard deviation, or as frequency and proportion (percentage), respectively. Normality and homogeneity were analyzed with Shapiro–Wilk and Levene tests. In turn, variables were compared between AD and HC using a two-sample t-test for normally distributed measures, or the Mann–Whitney U test for non-normally distributed measures. Relative abundance comparisons at the phylum, order, class, family, genus, and species levels were performed on normalized data employing non-parametric tests to detect differentially abundant features between the AD and HC groups. Richness (Chao1) and alpha-diversity (Shannon Index) metrics were calculated at the OTU level by performing rarefaction with 10 iterations of random subsampling to 1.788 reads (the lowest single-participant number of sequences) from each participant. In turn, independent two-sample t-tests for normally distributed measures or Mann–Whitney U tests for non-normally distributed measures in R were used. Beta-diversity metrics were computed using normalized OTU-level data in R, and included Bray–Curtis dissimilarity, Aitchison distance, and Jaccard similarity. To detect statistically significant differences in beta-diversity metrics between groups, a permutational multivariate analysis of variance (PERMANOVA) and analysis of similarity (ANOSIM) in the vegan package were used. The associations between GMB and cognitive, emotional, neuropsychiatric and functionality tests, as well as lifestyle factors for the AD group, were assessed using linear models with the Maaslin2 package. These associations included both richness and alpha-diversity, and relative abundance values at the phylum level. The default parameters of Maaslin2 were employed, with the addition of a minimum detection value and prevalence (0.2/100 and 50/100, respectively), in order to obtain more robust results. Significance for all tests was set at *p*-value < 0.05. To minimize false positives, a false discovery rate (FDR) correction (q-value < 0.1) was applied using the Benjamani–Hochberg method [38].

## 3. Results

### 3.1. Cohort Characteristics

Fifty participants met the eligibility criteria. They were classified as 25 individuals in the HC group and 25 individuals in the AD group. None of the groups differed in age, sex, or years of education (Table 1). The AD group did not show significant differences in Body Mass Index (BMI), smoking habits, alcohol consumption, or adherence to the MD compared to the HC group.

### 3.2. Cognitive Functions and Emotional, Neuropsychiatric, and Functionality Assessment

The cognitive functions and emotional, neuropsychiatric, and functional characteristics of the participants in the current study are shown in Table 2 and Table 3. Overall, the AD group exhibited significantly lower scores in the cognitive area, specifically in orientation, memory, attention, working memory, construction praxis, naming, fluency, and executive functions, compared to the HC group. These results indicate poorer performance in these areas by the AD group. In addition, significant differences were detected—compared to the HC group—in neuropsychiatric symptoms assessed by the NPBTII and NPEBTII tests, as well as in functionality, basic and instrumental activities of daily life, assessed using ADL test. Higher scores in these assessments correspond to greater impairment. Finally, no significant differences were observed between the two groups related to lifestyle, assessed with the LEQ test, or in the emotional area, assessed with the Goldberg Scale.

### 3.3. Alpha- and Beta-Diversity

**Figure 1:**
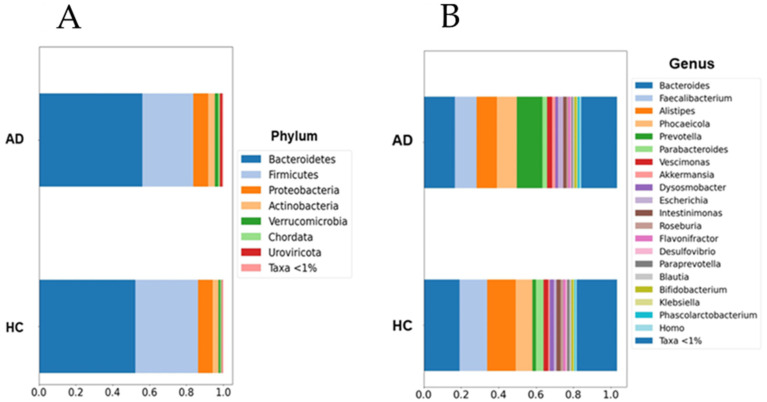
Barplots of microbial abundances at the phylum (**A**) and genus (**B**) levels. No significant differences were detected between groups. AD: Alzheimer’s disease; HC: healthy controls. Data are presented as percentages (0 to 1) of the mean relative abundances for each group.

**Figure 2:**
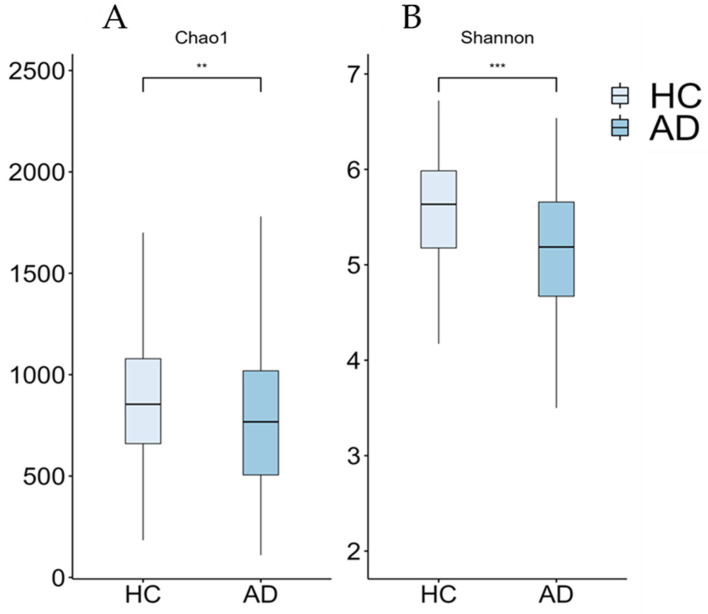
Boxplots of alpha-diversity measures (Chao1 (**A**) and Shannon (**B**) indices) from our cohort. Significant differences between groups are indicated by asterisks (** ≤0.01, *** ≤0.001). AD: Alzheimer’s disease; HC: healthy controls.

Although the current results suggest an increase in the abundance of the Prevotella and Escherichia genera within the AD group, this increase was not statistically significant when multiple non-parametric pairwise test were conducted (Figure 1). No significant differences were found across any of the other taxonomic ranks. However, richness (Chao1, *p* = 0.003) and alpha-diversity (Shannon Index, *p* < 0.001) were significantly lower in the AD group compared to the HC group (Figure 2). Furthermore, the PERMANOVA and ANOSIM tests of Bray–Curtis dissimilarity, Aitchison distance, and Jaccard distance did not show significant differences in beta-diversity between the two groups.

### 3.4. Diversity Measures and AD Cognitive Assessment

There were no associations between richness or alpha-diversity measures and cognitive functions.

### 3.5. Bacterial Phylum Abundance and AD Cognitive Assessment

The correlation between bacterial abundance at the phylum level and Mediterranean lifestyle, as well as the cognitive and functionality tests, is presented in Table 4 and Table 5. Only AD patients following the methodology applied in a previous study by Nuzum, Szymlek-Gay [39] were included. The linear models derived from Maaslin2 unveiled associations of 28 distinct phyla with orientation, activities of daily life (both basic and instrumental), and adherence to the MD, particularly in relation to food consumption.

## 4. Discussion

### 4.1. Cognitive Alterations

The cognitive decline that defines AD has been extensively studied for many years. This deterioration has been observed not only at the cognitive level, but also in the emotional, neuropsychiatric, and functional domains of patients affected by the disease [40,41,42]. If we focus on the cognitive domain, the AD patients of the present investigation exhibited deficiencies in orientation, memory, attention, working memory, construction praxis, naming, fluency, and executive functions compared to the HC group. These results are consistent with findings previously reported by other authors [41,43]. Furthermore, the AD group scored higher on tests on neuropsychiatric symptoms. This aligns with the scientific literature, indicating that AD patients tend to show more neuropsychiatric symptoms, including anxiety, depression, agitation, apathy, or sleep disturbances, due to the proliferation of amyloid plaques and phosphorylated tau protein, and the reduction in the hippocampal volume [42,44,45]. Regarding the assessment of functionality, the higher scores obtained by patients in the AD group indicate a higher degree of dependence in basic and instrumental activities of daily life. These findings were expected, considering that our patients were recruited with GDS4-5 scores, at a stage at which they required assistance from family members—or caregiving services—to adequately perform some of these tasks [46].

Finally, no significant differences between groups were found in relation to the emotional domain. Several studies have reported clear differences in this area [47,48]. These changes result from structural alterations in the brain, cognitive decline itself, and associated metabolic disruptions [49,50]. Anxiety is more prevalent in the early stages of AD, while depression becomes a more prominent feature in the later stages of the disease [41]. In the present study, emotionality was assessed using the Goldberg Scale [36] by asking the patients themselves. Additionally, the Neuropsychiatric Symptomatology from Barcelona Test II (NPBTII) [25] asked family members—or caregivers—about symptoms, and two of the items in this test asked about the patient’s anxiety and depression. The lack of significant differences observed in the Goldberg Scale could be attributed to the loss of insight often experienced by AD patients. As the disease progresses, many patients struggle to accurately assess their own emotional state, which can lead to a discrepancy between their self-reported answers and their actual condition. This limitation introduces a potential bias in the results, considering that the patients’ responses may not reflect their true emotional state, or being aligned with the observations made by their relatives or caregivers [51].

### 4.2. Alpha- and Beta-Diversity Differences among Groups

Regarding alpha-diversity indices, reductions in Chao1 and Shannon indices were observed in the AD group. The loss of alpha-diversity in AD patients is a consistent finding in most previous studies [9]. Table 6 summarizes the studies reviewed on the microbiota of AD and MCI patients compared with the current results. Vogt and Kerby [9] reported this phenomenon in the American population, obtaining the same reduction in the mentioned indices, as well as in Simpson and Inverse Simpson indices. Additionally, studies conducted in the Asian population showed the same trend [52,53,54]. Controversially, Saji and Miida [8] reported an increase in the Shannon Index and Simpson Index in Japanese AD patients, attributed to differences in diet and lifestyle.

The reduction in alpha-diversity has also been reported in other clinical conditions that are considered risk factors for the development of AD, such as obesity or diabetes [55,56]. Changes in microbial diversity may promote—through the gut–brain axis—the onset of inflammatory processes characteristic of these diseases [11].

However, alpha-diversity values vary among studies and diseases, leading some authors to question their utility to establish reliable diagnoses. The systematic review conducted by Plassais and Gbikpi-Benissan [57] highlighted this heterogeneity of results in studies involving Parkinson’s disease and Multiple Sclerosis patients. These authors did not find associations between richness and alpha-diversity values and the development of these neurodegenerative diseases (Parkinson’s disease and Multiple Sclerosis). Some limitations of that review included geographical differences in the analyzed populations, as well as variability in the alpha-diversity indices used in different studies. Nevertheless, their conclusions point to the need for standardizing analysis methods and accurately describing whether these indices are useful for diagnosing these diseases.

**Table 6:** Summary of Alzheimer’s disease studies with their most relevant goals compared with our results.

Reference	Number of Samples	Methods	Country	Results
This study	25 AD 25 HC	*Shotgun* metagenomics	Spain	↓ alpha-diversity (Chao1 and Shannon indices) Distinct microbial communities of AD compared with HC
Vogt et al., 2017 [9]	25 AD 25 HC	16S rRNA	United States	↓ alpha-diversity (Chao1, Shannon, Simpson, and Inverse Simpson indices) ↓ Firmicutes and Bifidobacterium ↑ Bacteroidetes
Haran et al., 2019 [54]	108 elders	*Shotgun* metagenomics	United States	↓ butyrate-producing taxa ↑ proinflammatory taxa
Saji, Niida et al., 2019 [8]	34 AD 94 HC	t-RFLP	Japan	↑ alpha-diversity (Shannon and Simpson indices) ↓ Bacteroides
Ueda et al., 2019 [50]	7 AD 15 MCI 21 HC	16S rRNA *Shotgun* metagenomics	Japan	↓ F. prausnitzii in MCI Abundances of this bacteria correlated with worse cognitive function
Guo et al., 2021 [48]	18 AD 20 MCI 18 HC	16S rRNA	China	Distinct microbial communities of AD compared with MCI and HC ↓ Bacteroides, Lachnospira, and Ruminiclostridium ↑ Prevotella Abundances correlated with worse cognitive function.
Liu et al., 2019 [49]	33 AD 32 MCI 32 HC	16S rRNA	China	Distinct microbial communities of AD compared with MCI and HC
Duan et al., 2021 [57]	18 MCI	16S rRNA	China	↓ Firmicutes ↑ Bacteroidetes
Pan et al., 2021 [55]	22 MCI 26 HC	16S rRNA	China	Distinct microbial communities of MCI compared with HC

↓: Decreases. ↑: Increases.

In the case of beta-diversity measures, no differences were observed between the AD group and the HC group. This lack of differences in the composition of the GMB differs from those described in AD [58]. Guo and Peng [52] emphasized beta-diversity changes in patients with AD and mild cognitive impairment (MCI), a prodromal state of the disease. Specifically, Pan and Li [59] were able to discriminate MCI patients and HC individuals into clusters, but they did not find differences in alpha-diversity measures. Therefore, changes in the community structures of the GMB may play a more relevant role than mere overall diversity loss.

Finally, no relationships were found between the different diversity indices and the studied variables. Some authors have emphasized that changes in the composition of the GMB are related to the concentrations of Aβ and Tau proteins, but not with neurodegeneration biomarkers [60]. This could indicate that although neurodegeneration does not occur as a direct consequence of changes in the GMB, these changes do play a role in the development of AD. Further studies, possibly involving early-stage AD or MCI patients, are clearly necessary to determine the effect of changes in the GMB on the development of AD.

### 4.3. Relation between Microbial Phylum Abundance and AD Cognitive Assessment

In the present study, differences in phylum-level abundance between the AD group and the HC group were investigated, but no significant results were obtained. Despite this, we have observed some trends similar to those described in previous studies, such as a reduction in Firmicutes and *Bacteroides* and an increase in Bacteroidetes [60]. The decrease in Firmicutes and *Bifidobacterium* and the increase in Bacteroidetes have been reported in both American and Chinese AD patients [9,61]. Additionally, the same changes have been observed in conditions considered risk factors for AD, such as diabetes and obesity [55,56]. Bacteroidetes has been associated with inflammatory processes, while Firmicutes and *Bifidobacterium* tend to be associated with anti-inflammatory processes, highlighting the importance of these processes in the development of AD [9,62]. In the current investigation, the abundance of *Bifidobacterium* remained stable in both groups. Interestingly, in a study conducted in Japanese AD patients, a reduction in *Bacteroides* was found [8]. The same research group emphasized the role of *Bacteroides* as a protective agent against cognitive deficits [63]. This genus belongs to the Bacteroidetes, but it is known for its anti-inflammatory properties. Our sample shows a slight decrease in *Bacteroides*, without reaching statistical significance. The discrepancies between the results could be explained by differences in diet and lifestyle across different regions/countries. The dietary pattern of the Western diet is characterized by a high consumption of saturated fats and carbohydrates, unlike the MD, which is characterized by an abundance of plant-based foods and healthy fats [64,65].

Other observations included the non-significant increase in *Escherichia* in the AD group. Cattaneo and Cattane [62] described a significant increase in this genus, highlighting that its higher abundance favored the development of inflammatory processes in Italian AD patients. Similar results were also obtained in Thai AD patients [66]. Moreover, fragments of *E. coli* were found in the brains of AD patients, co-localizing with amyloid plaques, suggesting that it could be promoting amyloidosis through an inflammatory state [67].

Furthermore, linear models conducted in RStudio resulted in associations of different phyla with three groups of variables: orientation, adherence to the MD (specifically in food consumption), and activities of daily life (see Table 7). In the case of adherence to the MD, a negative correlation was found with Proteobacteria levels. This Gram-negative bacterial phylum has been associated with various inflammatory diseases. Thus, the higher adherence to the MD would be acting as a protective factor against these inflammatory processes [65,68]. On the other hand, we found non-significant low levels of Firmicutes in AD patients, positively correlated with reductions in instrumental activities of daily life. The reduction in Firmicutes has been reported in AD studies [9,61]. Controversially, in a study with PD, elevated levels of Firmicutes were found, associated with indicators of inflammation and immune system dysregulation [69]. Therefore, their role in AD requires further investigations.

### 4.4. Strengths and Limitations

The strengths of the present study include the combination of a metagenomic methodology along with a comprehensive neuropsychological battery of tests, which allow a thorough characterization of our patients, facilitating the identification of associations between symptomatology and changes in the microbiota. However, our study also has also several limitations. The number of patients was relatively low and confined to our geographic region. Consequently, caution must be exercised when extrapolating the current results. Characterizing dietary patterns would require more specific targeted tests in order to control their effects on intestinal microbial populations. Finally, to the best of our knowledge, there are no normative data available for the adult population regarding the MEDLIFE questionnaire. This lack of data makes it difficult to determine whether the sample is representative of the general Spanish population.

## 5. Conclusions

The current investigation has shown that richness and alpha-diversity are decreased in Spanish AD patients. Additionally, discrete relationships were found between various phyla and orientation, activities of daily life, and adherence to the MD. To the best of our knowledge, this is the first study aimed at proposing a multidisciplinary approach considering microbiological, psychological, and lifestyle parameters. Further investigations conducted in other countries with a Mediterranean lifestyle are required to confirm the results of the present study.

## Figures and Tables

**Table 1:** Cohort characteristics by group.

	HC (*n* = 25)		AD (*n* = 25)			*p*-Value
	Mean or Count	SD or %	Mean or Count	SD or %		
Age (years)	70.6	4.9	73.0	5.0	t(48) = 1.71	0.090
Sex (M/F)	13M, 12F	52% M	12M, 13F	48% M	Χ^2^ = 0.08	0.780
BMI (kg/m^2^)	26.6	3.2	24.7	3.6	t(48) = 1.96	0.060
Education (years)	10.4	4.8	9.8	5.0	t(48) = 0.43	0.670
Smoke status						
Current smoker	3	12%	1	4%	Χ^2^ = 1.13	0.570
Former smoker	8	32%	8	32%		
Non-smoker	14	56%	16	64%		
Alcohol consumption						
Current consumer	7	28%	3	12%	Χ^2^ = 3.70	0.150
Former consumer	0	0%	2	8%		
Non-consumer	18	72%	20	80%		
MEDLIFE						
Food consumption	9.80	1.63	10.20	1.63	F(1,48) = 0.75	0.391
Dietary habits	4.60	1.08	4.40	1.15	F(1,48) = 0.64	0.421
Physical and social activity	3.88	0.97	3.44	1.04	F(1,48) = 2.13	0.144

Displayed as mean and standard deviation for continuous data, and as count and percentage for categorical data. AD: Alzheimer’s disease; BMI: Body Mass Index; F: female; HC: healthy controls; M: male; MEDLIFE: Mediterranean Lifestyle Index Interview; SD: standard deviation.

**Table 2:** Cognitive and emotional characteristics by group.

	HC		AD			*p*-Value
	Mean or Count	SD or %	Mean or Count	SD or %		
OBTII						
Personal orientation	25.00	0.00	23.60	2.24	H(1) = 12.06	**<0.001**
Spatial orientation	24.60	2.00	20.28	5.79	H(1) = 23.38	**<0.001**
Temporary orientation	69.60	2.00	46.32	23.34	H(1) = 27.73	**<0.001**
MMSE	28.88	1.79	21.72	4.30	F(1,48) = 59.18	**<0.001**
MIS	6.83	1.40	1.64	2.12	H(1) = 30.83	**<0.001**
DS						
Forward span	5.60	1.22	4.80	1.08	H(1) = 4.60	**0.032**
Backward span	3.84	1.03	3.48	0.96	H(1) = 1.52	0.217
FCSRT						
1st free recall	5.40	2.40	0.84	1.25	t(48) = 8.42	**<0.001**
Total free recall	20.12	8.04	2.28	5.28	H(1) = 31.56	**<0.001**
Total recall	37.16	10.11	5.64	11.87	H(1) = 32.72	**<0.001**
Delayed free recall	8.32	3.78	0.20	0.65	H(1) = 36.51	**<0.001**
Total delayed recall	13.20	3.71	1.36	2.97	H(1) = 35.18	**<0.001**
TMT A	61.64	35.72	218.00	268.36	H(1) = 8.24	**0.004**
TMT B	201.17	262.30	225.00	330.92	H(1) = 0.88	0.346
CDT	9.08	1.76	5.62	3.35	H(1) = 15.07	**<0.001**
PCBTII	29.20	2.06	26.68	4.79	H(1) = 5.08	**0.024**
FAB	17.08	1.11	12.24	4.63	H(1) = 19.45	**<0.001**
CRS	12.40	5.42	11.12	4.51	F(1,48) = 0.82	0.369
Boston-C	12.00	1.71	9.40	3.13	t(37.1) = −3.64	**<0.001**
CEF						
Semantic fluency	16.87	4.66	10.28	4.79	F(1,47) = 23.80	**<0.001**
Formal fluency	12.68	5.86	8.44	4.91	F(1,48) = 7.69	**0.008**
GOLDBERG						
Anxiety scale	1.32	2.32	1.12	1.83	H(1) = 0.04	0.826
Depression scale	0.92	1.87	0.96	2.01	H(1) = 0.04	0.840
LEQ	12.48	5.22	10.79	5.30	F(1,47) = 1.26	0.267

Displayed as mean and standard deviation for continuous data, and as count and percentage for categorical data. Bold indicates significant differences in the statistical test. AD: Alzheimer’s disease; Boston-C: Abbreviated Boston Naming Test version C; CDT: Clock Drawing Test; CEF: Categorial Evocation Fluency from Barcelona Test II; CRS: Cognitive Reserve Scale; DS: Digit Span from Barcelona Test II; FAB: Frontal Assessment Battery; FCSRT: Free and Cued Selective Reminding Test; GOLDBERG: Anxiety and Depression Scale; HC: healthy control; LEQ: Life Events Questionnaire; MIS: Memory Impairment Screen; MMSE: Mini Mental Score Examination; OBTII: Orientation from Barcelona Test II; PCBTII: Construction Praxis from Barcelona Test II; SD: standard deviation; TMT A: Trail Making Test A; TMT B: Trail Making Test B.

**Table 3:** Neuropsychological and functionality characteristics by group.

	HC		AD			*p*-Value
	Mean or Count	SD or %	Mean or Count	SD or %		
NPBTII	1.04	2.99	5.96	4.20	H(1) = 25.59	**<0.001**
NPEBTII	0.00	0.00	0.42	0.88	H(1) = 5.66	**0.017**
ADL						
Basic activities	0.00	0.00	1.88	3.44	H(1) = 13.58	**<0.001**
Instrumental activities	0.20	0.71	32.44	15.60	H(1) = 40.34	**<0.001**

Displayed as mean and standard deviation for continuous data, and as count and percentage for categorical data. Bold indicates significant differences in statistical tests. AD: Alzheimer’s disease; ADL: Activities of Daily Living from Barcelona Test II; HC: healthy control; NPEBTII: Complementary Neuropsychiatric Symptomatology from Barcelona Test II; NPBTII: Neuropsychiatric Symptomatology from Barcelona Test II; SD: standard deviation.

**Table 4:** Association between Mediterranean lifestyle and relative microbial abundance in Alzheimer’s disease group.

FEATURE	METADATA	COEF	StdErr	N	N not 0	*p*	*q*
Phylum.Acidobacteria	MEDcT	−0.37471	0.12475	25	25	0.006	0.036
Phylum.Acidobacteria	MEDT	−0.22184	0.07320	25	25	0.006	0.061
Phylum.Actinobacteria	MEDcT	−0.33567	0.13587	25	25	0.021	0.066
Phylum.Armatimonadetes	MEDcT	−0.45215	0.13859	25	22	0.003	0.036
Phylum.Armatimonadetes	MEDT	−0.22803	0.08638	25	22	0.015	0.062
Phylum.Chlorobi	MEDcT	−0.33536	0.12306	25	25	0.012	0.046
Phylum.Chlorobi	MEDT	−0.19716	0.07240	25	25	0.012	0.061
Phylum.Chloroflexi	MEDcT	−0.36241	0.12745	25	25	0.009	0.044
Phylum.Chloroflexi	MEDT	−0.20554	0.07589	25	25	0.013	0.061
Phylum.Cyanobacteria	MEDT	−0.14261	0.05837	25	25	0.023	0.070
Phylum.Deinococcus.Thermus	MEDcT	−0.42398	0.15167	25	25	0.010	0.044
Phylum.Deinococcus.Thermus	MEDT	−0.23198	0.09124	25	25	0.018	0.065
Phylum.Gemmatimonadetes	MEDcT	−0.46382	0.15229	25	25	0.006	0.036
Phylum.Gemmatimonadetes	MEDT	−0.27341	0.08951	25	25	0.006	0.061
Phylum.Kiritimatiellaeota	MEDcT	−0.53515	0.16450	25	24	0.004	0.036
Phylum.Kiritimatiellaeota	MEDT	−0.27882	0.10145	25	24	0.011	0.061
Phylum.Nitrospirae	MEDcT	−0.36010	0.14063	25	25	0.017	0.059
Phylum.Nitrospirae	MEDT	−0.20941	0.08299	25	25	0.019	0.065
Phylum.Omnitrophica	MEDcT	−0.45225	0.19750	25	17	0.032	0.089
Phylum.Omnitrophica	MEDT	−0.27288	0.11549	25	17	0.027	0.076
Phylum.Planctomycetes	MEDcT	−0.35369	0.11748	25	25	0.006	0.036
Phylum.Planctomycetes	MEDT	−0.20408	0.06962	25	25	0.008	0.061
Phylum.Proteobacteria	MEDT	−0.25193	0.05843	25	25	<0.001	0.009

COEF: Coefficient; StdErr: standard error; *p*: statistical significance; *q*: statistical significance with false discovery rate (FDR) correction; MEDcT: MEDLIFE food consumption; MEDT: MEDLIFE total score.

**Table 5:** Association between cognitive assessment and relative microbial abundance in Alzheimer’s disease group.

FEATURE	METADATA	COEF	StdErr	N	N not 0	*p*	*q*
Phylum.Thermodesulfobacteria	POBTII	0.32178	0.83389	25	24	0.001	0.044
Phylum.Thermodesulfobacteria	SOBTII	0.31115	0.83137	25	24	0.001	0.044
Phylum.Thermodesulfobacteria	TOBTII	0.31320	0.83108	25	24	0.001	0.044
Phylum.Thermodesulfobacteria	OBTII	−0.31225	0.82957	25	24	0.001	0.044
Phylum.Acidobacteria	ADLTBTII	−0.20988	0.06986	25	25	0.007	0.056
Phylum.Acidobacteria	ADLIBTII	0.23272	0.07885	25	25	0.007	0.056
Phylum.Aquificae	ADLTBTII	−0.17086	0.06520	25	25	0.016	0.056
Phylum.Aquificae	ADLIBTII	0.19350	0.07359	25	25	0.015	0.056
Phylum.Armatimonadetes	ADLIBTII	0.18689	0.09763	25	22	0.069	0.094
Phylum.Bacteroidetes	ADLIBTII	−0.06670	0.03297	25	25	0.055	0.088
Phylum.Calditrichaeota	ADLTBTII	−0.23865	0.08102	25	21	0.007	0.056
Phylum.Chrysiogenetes	ADLTBTII	−0.20443	0.08632	25	23	0.027	0.056
Phylum.Chrysiogenetes	ADLIBTII	0.22331	0.09742	25	23	0.032	0.060
Phylum.Cyanobacteria	ADLBBTII	−0.14739	0.04294	25	25	0.002	0.077
Phylum.Cyanobacteria	ADLTBTII	−0.17467	0.04975	25	25	0.002	0.053
Phylum.Cyanobacteria	ADLIBTII	0.18648	0.05615	25	25	0.003	0.053
Phylum.Deferribacteres	ADLTBTII	−0.15443	0.06382	25	25	0.024	0.056
Phylum.Deferribacteres	ADLIBTII	0.17102	0.07204	25	25	0.027	0.056
Phylum.Deinococcus.Thermus	ADLTBTII	−0.19024	0.09019	25	25	0.047	0.079
Phylum.Fibrobacteres	ADLIBTII	0.20090	0.09244	25	23	0.041	0.071
Phylum.Firmicutes	ADLTBTII	−0.10671	0.04554	25	25	0.029	0.057
Phylum.Firmicutes	ADLIBTII	0.11630	0.05140	25	25	0.034	0.061
Phylum.Fusobacteria	ADLTBTII	−0.15690	0.06588	25	25	0.026	0.056
Phylum.Fusobacteria	ADLIBTII	0.18415	0.07436	25	25	0.021	0.056
Phylum.Gemmatimonadetes	ADLTBTII	−0.21647	0.08996	25	25	0.025	0.056
Phylum.Gemmatimonadetes	ADLIBTII	0.23129	0.10154	25	25	0.033	0.060
Phylum.Kiritimatiellaeota	ADLTBTII	−0.25375	0.09809	25	24	0.017	0.056
Phylum.Kiritimatiellaeota	ADLIBTII	0.28087	0.11071	25	24	0.019	0.056
Phylum.Nitrospirae	ADLTBTII	−0.19524	0.07960	25	25	0.023	0.056
Phylum.Nitrospirae	ADLIBTII	0.21528	0.08984	25	25	0.026	0.056
Phylum.Omnitrophica	ADLTBTII	−0.33695	0.09838	25	17	0.002	0.053
Phylum.Omnitrophica	ADLIBTII	0.39218	0.11104	25	17	0.002	0.053
Phylum.Planctomycetes	ADLTBTII	−0.18046	0.06805	25	25	0.015	0.056
Phylum.Planctomycetes	ADLIBTII	0.19990	0.07681	25	25	0.016	0.056
Phylum.Spirochaetes	ADLTBTII	−0.12290	0.05052	25	25	0.024	0.056
Phylum.Spirochaetes	ADLIBTII	0.14160	0.05702	25	25	0.021	0.056
Phylum.Synergistetes	ADLTBTII	−0.27061	0.11414	25	25	0.027	0.056
Phylum.Synergistetes	ADLIBTII	0.30977	0.12883	25	25	0.025	0.056
Phylum.Thermotogae	ADLTBTII	−0.15337	0.06481	25	25	0.027	0.056
Phylum.Thermotogae	ADLIBTII	0.16936	0.07315	25	25	0.030	0.059

COEF: Coefficient; StdErr: standard error; *p*: statistical significance; *q*: statistical significance with false discovery rate (FDR) correction; POBTII: orientation to person (Barcelona test II); SOBTII: orientation to space (Barcelona test II); TOBTII: orientation to time (Barcelona test II); OBTII: orientation (total score—Barcelona test II); ADLTBTII: activities of daily living (total score—Barcelona test II); ADLIBTII: instrumental activities of daily living (Barcelona test II); ADLBBTII: basic activities of daily living (Barcelona test II).

**Table 7:** Summary of the significant associations observed in the present study between microbial phyla, adherence to the Mediterranean lifestyle, and neurocognitive outcomes.

Medyterranean Style Index (MEDLIFE)	Activities of Daily Living	Orientation
Phylum.Acidobacteria	Phylum.Acidobacteria	Phylum.Thermodesulfobacteria
Phylum.Actinobacteria	Phylum.Aquificae	
Phylum.Armatimonadetes	Phylum.Armatimonadetes	
Phylum.Chlorobi	Phylum.Bacteroidetes	
Phylum.Chloroflexi	Phylum.Calditrichaeota	
Phylum.Cyanobacteria	Phylum.Chlamydiae	
Phylum.Deinococcus.Thermus	Phylum.Chlorobi	
Phylum.Gemmatimonadetes	Phylum.Chloroflexi	
Phylum.Kiritimatiellaeota	Phylum.Chrysiogenetes	
Phylum.Nitrospirae	Phylum.Cyanobacteria	
Phylum.Omnitrophica	Phylum.Deferribacteres	
Phylum.Planctomycetes	Phylum.Deinococcus.Thermus	
Phylum.Proteobacteria	Phylum.Fibrobacteres	
	Phylum.Firmicutes	
	Phylum.Fusobacteria	
	Phylum.Gemmatimonadetes	
	Phylum.Kiritimatiellaeota	
	Phylum.Nitrospirae	
	Phylum.Omnitrophica	
	Phylum.Planctomycetes	
	Phylum.Spirochaetes	
	Phylum.Synergistetes	
	Phylum.Thermotogae	

## Data Availability

Dataset available on request from the authors.

## Supplementary Materials

The following supporting information can be downloaded at https://www.mdpi.com/article/10.3390/microorganisms12102046/s1, Table S1: Description of the tests/scales used in the study.
