# Supplementary material for: Gut Microbiota Alterations in Alzheimer’s Disease: Relation with Cognitive Impairment and Mediterranean Lifestyle
Source: Microorganisms. 2024 Oct 10;12(10):2046. doi: 10.3390/microorganisms12102046 (PMC11510339; doi:10.3390/microorganisms12102046)
Supplement: Supplementary file 1 [file microorganisms-12-02046-s001.zip › microorganisms-3253445-supplementary.pdf]

SUPPLEMENTARY MATERIAL

Table S1

TEST	DESCRIPTION
Temporary, Spatial and Personal Orientation (Barcelona-2 Test) [25]	Participants were assessed on their orientation into three areas: *personal orientation*, *spatial orientation,* and *time orientation*. This subtest measured orientation and other cognitive abilities, such as memory (short and long term), perception, attention, recognition of the environment, and the ability to integrate it. The total score ranged from 0 to 120 (personal orientation 0 - 25 + spatial orientation 0 - 25 + time orientation 0 -70).
Mini-Mental State Examination [26]	Participants were assessed on their general cognitive function, including orientation, attention, memory, language, calculation, comprehension, naming, abstraction, writing and drawing. The total score was 30.
Memory Impairment Screen [27]	Participants were assessed on their ability to recall four words that they had previously read. The examiner also provided categorical association for these words. The score was calculated as (free recall x 2) + facilitated recall + 1 (if they were 76 years).
Digit Span (Barcelona-2 Test) [25]	*Forward digit*: Participants were asked to repeat a series of digits. Measured their immediate attention, vigilance, and immediate memory capacity. *Backward digit*: Participants were asked to repeat the digits in reverse order. Measured immediate attention span, immediate memory, and active processing capacity.
Free and Cued Selective Reminding Test [28]	Participants were asked to be remembered sixteen words. The examiner also provided sixteen words for them to form categorical association. The test consisted of three trials and a delayed trial. In each trial, participants were assessed on their free call and provided with semantic cue. Measured verbal learning, short- and long-term memory, and selective reminding.
Trail Making Test A and B [29]	*Part A*: Participants were asked to connect the circles numbered 1 to 25 in numerical sequence. Measured their graphomotor speed, visual scanning, and executive function, as planification, flexibility cognitive. *Part B*: Participants were asked to connect numerical circles (1 to 13) with letters (A to L) while alternating between numbers and letters in an ascending sequence (1-A-2). Measured divided attention, mental flexibility, executive functions such as inhibition control and working memory.
Clock Drawing Test (*Cacho et. al* correction) [30]	Participants were asked to draw a clock with specific details. This test measured cognitive abilities, including verbal comprehension, executive functions (planning), visual memory and visual-spatial skills, motor programming and execution, numerical knowledge, abstract thought, and inhibitory control. The total score of this test is 10 (sphere 0 - 2 + hands 0 - 4 + numbers 0 - 4).

Construction Praxis (Barcelona-2 Test) [25]	Participants were asked to copy figures. This test measure executive functions such as planning and execution, visual-spatial and motor coordination, motor action skills and visual-perceptive recognition. For more complex figures, it also involved constructive problem-solving abilities. The total score of this test is 30 (0 - 3 for each teen draw).
Frontal Assessment Battery [32]	Participants were asked to be performed various tasks. *Conceptualization*: to say resemblance between two, for example, a banana and an orange. *Lexical Fluency*: produce as many words as possible. *Sequences*: memorize and repeat three hand movements: knuckle, chant, slap. *Sensitivity to interference and inhibitory control*: A commands were given to them. For example, when a tap was given by the examiner, they responded with two taps, and then the series following the given commands had to be performed by them. *Autonomy of the environment*: The examiner moved their hands towards the participant's hands. If the participant took the examiner's hands, the examiner would say, "Don't hold my hands now" and bring their hands back together. This test measured global executive dysfunction, specifically frontal lobe dysfunction, including impulse and inhibitory control, mental flexibility, and sensitivity to interference. The total score of this test is 18 (0 - 3 for each task).
Cognitive Reserve Scale [33]	Participants were asked about their education, occupation, and lifestyle factors. This scale measures the level of activity of the participant, both physical and cognitive. The total score was 25.
Abbreviated Boston Naming Test version C [34]	Participants were asked to name the 15 objects depicted. Measures the ability to name objects and specifically aimed to evaluate anomia, which refers to the impairment in the ability to recall and name objects. The total score was 15, one point for each answer without phonemic cue.
Categorial Evocation Fluency [25]	*Semantic fluency task*: Participants were asked to generate words belonging to the category of "animals" for one minute. Measure language skills, semantic memory, semantic verbal fluency, working memory, and executive function like inhibitory control. *Formal lexical task*: Participants were asked to generate words for one minute. Measure phonemic verbal fluency, language skills, working memory and executive function, including inhibitory control.
Anxiety and Depression Scale - Goldberg [36]	Participants were asked questions about symptoms of anxiety or depression. It contains two subscales with nine questions each: the anxiety subscale (questions 1–9) and the depression subscale (questions 10–18). The first four questions of each subscale serve as a screening criterion to determine if the rest of the questions should be attempted. Specifically, if fewer than two of the first four questions are answered affirmatively, the remaining questions of the anxiety subscale should not be answered. In the case of the depression subscale, answering at least one of the first four questions affirmatively is sufficient to proceed with the rest of the questions. This scale measured symptomatology related to

	anxiety and depression. The total score was 18, one for each question.
Life Events Questionnaire [37]	Participants were asked questions regarding events that had occurred throughout their lives. They were required to assess the degree of emotional impact produced by each event. This test measured the perception of stress events.
Mediterranean Lifestyle Index Interview [21]	Participants were asked about the frequency of consuming various foods. The questionnaire aimed to assess adherence to a healthy Mediterranean lifestyle. MEDLIFE was divided into three indices: *consumption*, *habit*, and *activity*, incorporation traditional healthy lifestyle and cultural elements, such as physical and social activities or time to rest. The total score was 28 (15 consumption index + 7 habit index + 6 activity index).
Neuropsychiatric Symptomatology (Barcelona-2 Test) [25]	Family members were asked about neuropsychiatric symptoms (21 items). It measures the presence and severity symptoms such as depression, anxiety, apathy, disinhibition, among many others. There was also a complementary questionnaire (7 items) addressing specific neuropsychiatric symptoms, including anosognosia and optic ataxia, etc. The total score was 63 (0 - 3 for each question), and 21 for the complementary questionnaire (0 - 3 each question).
Activities of Daily-Living (Barcelona-2 Test) [25]	Family members were asked to provided information on any difficulties or inability to perform these activities. The test encompassed three categories: *basic activities*, *instrumental activities,* and *advanced activities*. This test measures the functional abilities of the participant and their dependency level of that independent in daily activities. The total score was 100 (60 for advanced and instrumental activities + 40 for basic activities).
